# Structure of New Ferroverdins Recruiting Unconventional Ferrous Iron Chelating Agents

**DOI:** 10.3390/biom12060752

**Published:** 2022-05-26

**Authors:** Loïc Martinet, Dominique Baiwir, Gabriel Mazzucchelli, Sébastien Rigali

**Affiliations:** 1 InBioS, Center for Protein Engineering, University of Liege, B-4000 Liege, Belgium; 2 Hedera-22, Boulevard du Rectorat 27b, B-4000 Liege, Belgium; loic@hedera22.com; 3 GIGA Proteomics Facility, University of Liege, B-4000 Liege, Belgium; D.Baiwir@uliege.be; 4 Mass Spectrometry Laboratory, MolSys Research Unit, University of Liege, B-4000 Liege, Belgium; gabriel.mazzucchelli@uliege.be

**Keywords:** CETP inhibitors, iron complexes, Streptomyces, HDL cholesterol, metal-nitrosophenolato compounds, natural products, biosynthetic gene cluster

## Abstract

Ferroverdins are ferrous iron (Fe^2+^)-nitrosophenolato complexes produced by a few *Streptomyces* species as a response to iron overload. Previously, three ferroverdins were identified: ferroverdin A, in which three molecules of *p*-vinylphenyl-3-nitroso-4-hydroxybenzoate (*p*-vinylphenyl-3,4-NHBA) are recruited to bind Fe^2+^, and Ferroverdin B and Ferroverdin C, in which one molecule of *p*-vinylphenyl-3,4-NHBA is substituted by hydroxy-*p*-vinylphenyl-3,4-NHBA, and by carboxy-*p*-vinylphenyl-3,4-NHBA, respectively. These molecules, especially ferroverdin B, are potent inhibitors of the human cholesteryl ester transfer protein (CETP) and therefore candidate hits for the development of drugs that increase the serum concentration of high-density lipoprotein cholesterol, thereby diminishing the risk of atherosclerotic cardiovascular disease. In this work, we used high-resolution mass spectrometry combined with tandem mass spectrometry to identify 43 novel ferroverdins from the cytosol of two *Streptomyces lunaelactis* species. For 13 of them (designated ferroverdins C2, C3, D, D2, D3, E, F, G, H, CD, DE, DF, and DG), we could elucidate their structure, and for the other 17 new ferroverdins, ambiguity remains for one of the three ligands. *p*-formylphenyl-3,4-NHBA, *p*-benzoic acid-3,4-NHBA, 3,4-NHBA, *p*-phenylpropionate-3,4-NHBA, and *p*-phenyacetate-3,4-NHBA were identified as new alternative chelators for Fe^2+^-binding, and two compounds (C3 and D3) are the first reported ferroverdins that do not recruit *p*-vinylphenyl-3,4-NHBA. Our work thus uncovered putative novel CETP inhibitors or ferroverdins with novel bioactivities.

## 1. Introduction

**Figure 1:**
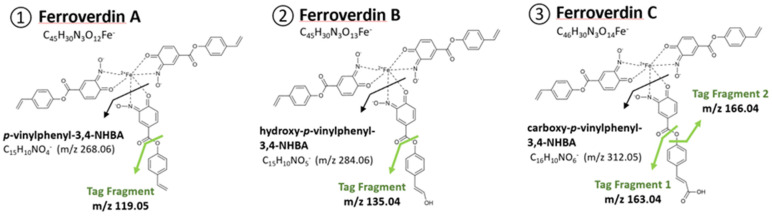
**Molecular tag signals for the identification of ferroverdin-related compounds.** ①For ferroverdin A, B, and C, the *m*/*z* ratios are 268.06 and 119.05 (for the *p*-vinylphenyl-3,4-NHBA and its major MS fragment); ②For ferroverdin B, the *m*/*z* ratios are 284.06 and 135.04 (for the hydroxy-*p*-vinylphenyl-3,4-NHBA and its major MS fragment); ③ For ferroverdin C, the *m*/*z* ratios are 312.05, 163.04, and 166.04 (for carboxy-*p*-vinylphenyl-3,4-NHBA and its two major MS fragments).

Ferroverdins, together with the antibiotics viridomycins and actinoverdins, are green-pigmented ferrous (iron(II)) ion (Fe^2+^)-nitrosophenolato complexes [1] produced by a few members of the *Streptomyces* genus. Ferroverdins A, B, and C were originally isolated from the fermentation broth of *Streptomyces* WK-5344 [2] and later identified as main compounds produced by the cave-moonmilk-dwelling species *Streptomyces lunaelactis* [3,4,5,6,7]. In ferroverdin A, Fe^2+^ is bound by three *p*-vinylphenyl-3-nitroso-4-hydroxybenzoate (*p*-vinylphenyl-3,4-NHBA) molecules (Figure 1, compound 1) [8,9]. In ferroverdin B and ferroverdin C, hydroxy-*p*-vinylphenyl-3,4-NHBA and carboxy-*p*-vinylphenyl-3,4-NHBA substitute one molecule of *p*-vinylphenyl-3,4-NHBA, respectively (Figure 1, compounds 2 and 3) [8,9,10].

The condition for the intracellular accumulation of ferroverdins by streptomycetes contrasts with the one that favors the secretion of siderophores [11]. Indeed, while the production of the latter is triggered upon iron depletion in order to capture environmental ferric (iron(III)) ions (Fe^3+^) and subsequent uptake, ferroverdin production is instead activated upon iron overload [4,11,12]. The importance of the secreted siderophores is well known in streptomycetes, playing crucial roles for housekeeping functions, survival under microbial competition and in an iron-depleted environment [13], sporulation [13,14], chemical differentiation [13,14,15,16,17,18,19], and possibly in programmed cell death [19,20]. In contrast, the physiological role of ferroverdins for the producing microorganism is currently unknown.

The synthesis of ferroverdins also presents a unique feature as it depends on a biosynthetic gene cluster (*fev*/*bag*) also involved in the production of the amino–aromatic antibiotics called bagremycins [12,21]. Bagremycins result from the condensation of 3-amino-4-hydroxybenzoic acid (3,4-AHBA) with *p*-vinylphenol by the bagremycin synthetase FevW/BagE [12,22]. When iron is abundant, FevW additionally uses the substrate 4-hydroxy-3-nitrosobenzoic acid (3,4-NHBA) for condensation with *p*-vinylphenol, which results in the production of *p*-vinylphenyl-3,4-NHBA, the chelating agent primarily recruited for binding Fe^2+^ in the three known ferroverdins. The *bag*/*fev* cluster is thus a unique example of a biosynthetic gene cluster involved in the production of two structurally diverse molecules with different bioactivities [12].

Although the biological role of ferroverdins remains to be discovered, these molecules are known, potent inhibitors of the human cholesteryl ester transfer protein (CETP) [23,24]. CETP transfers cholesteryl esters from non-atherogenic, high-density lipoproteins (HDL) to potentially proatherogenic, low-density lipoprotein (LDL) fractions. Inhibitors of CETP thus increase the concentration of HDL cholesterol and decrease LDL cholesterol concentration, which is predicted to reduce cardiovascular disease risk. Finding inhibitors of CETP to raise HDL cholesterol levels is still regarded as a possible strategy for reducing cardiovascular events, despite three compounds having failed in phase III clinical trials [25,26,27,28].

In this work, we reveal how the combination of the analysis of the MS-based-fragmentation, molecular-tagging patterns with the specific ^54^Fe/^56^Fe isotope ratio distribution allowed the identification of 46 novel ferroverdins from the crude extracts of two *Streptomyces lunaelactis* strains (strains MM37 and MM109^T^). For 13 of these new ferroverdins, we could elucidate their structure and identify novel molecules that participate in the chelation of the ferrous ion, thereby highlighting putative novel CETP-inhibitors or ferroverdins with novel bioactivities.

## 2. Materials and Methods

### 2.1. Strains and Culture Conditions

*S*. *lunaelactis* MM37 and MM109^T^ strains were cultured in the R2YE medium [29] supplemented with 1 mM FeCl_3_ in order to induce the production of ferroverdins as described previously [12].

### 2.2. Compound Identification

Extracts were analyzed by ultra-performance liquid chromatography–tandem mass spectrometry (Acquity UPLC I-Class, Waters—Q Exactive Plus, Thermo Fisher Scientific). Each compound was identified according to its exact mass (mass tolerance < 5 ppm), the isotopic pattern, the MS/MS spectra of the molecular ion HCD fragmentation, and the UV–Vis absorbance spectra. The detailed protocols for ferroverdin extraction and identification are described in [4]. For the analysis of the MS-based-fragmentation, molecular-tagging patterns, the ferroverdin monomer fragments were searched, allowing a mass tolerance of < 3 ppm, and were fixed as the main intensity peak of the fragmentation spectra (intensity = 100%). The tag fragments of each ferroverdin monomer (Figure 1) were searched, allowing a mass tolerance of < 5 ppm, and present a peak intensity of > 7.5%, compared to the intensity of their respective ferroverdin monomer fragments (*p*-vinylphenyl-3,4-NHBA, hydroxy-*p*-vinylphenyl-3,4-NHBA, and carboxy-*p*-vinylphenyl-3,4-NHBA).

## 3. Results

The crude extract of the two strains of *S*. *lunaelactis,* MM37 and MM109^T^, grown on solid R2YE medium supplemented with 1 mM FeCl_3_*,* were analyzed by UPLC–MS/MS. Ions corresponding to ferroverdins were identified by searching for the presence of an iron atom, which can be inferred from mass spectra due to the specific isotopic distribution of naturally occurring stable isotopes: ^54^Fe (5.845%), ^56^Fe (91.754%), ^57^Fe (2.119%), and ^58^Fe (0.286%). Classically, the iron signature in molecules is indicated by a 1.995 Da difference between isotopic ^54^Fe and ^56^Fe signals. The M-2 peak (^54^Fe) has a relative intensity corresponding to ~6.4% of the intensity of the M peak (^56^Fe) This strategy, based on isotope-assisted screening for iron-containing metabolites combined with high-resolution LCMS, has previously been used with success to identify siderophores and other iron-binding chelators [30,31,32,33]. In addition, we used a series of molecular tags in the fragmentation pattern to discriminate ferroverdin-like compounds from other iron-containing molecules. Figure 1 shows the molecular tag signals that can be obtained from the fragmentation of the molecules involved in ferrous iron chelation in ferroverdins, i.e., *p*-vinylphenyl-3,4-NHBA, hydroxy-*p*-vinylphenyl-3,4-NHBA, and carboxy-*p*-vinylphenyl-3,4-NHBA.

**Figure 2:**
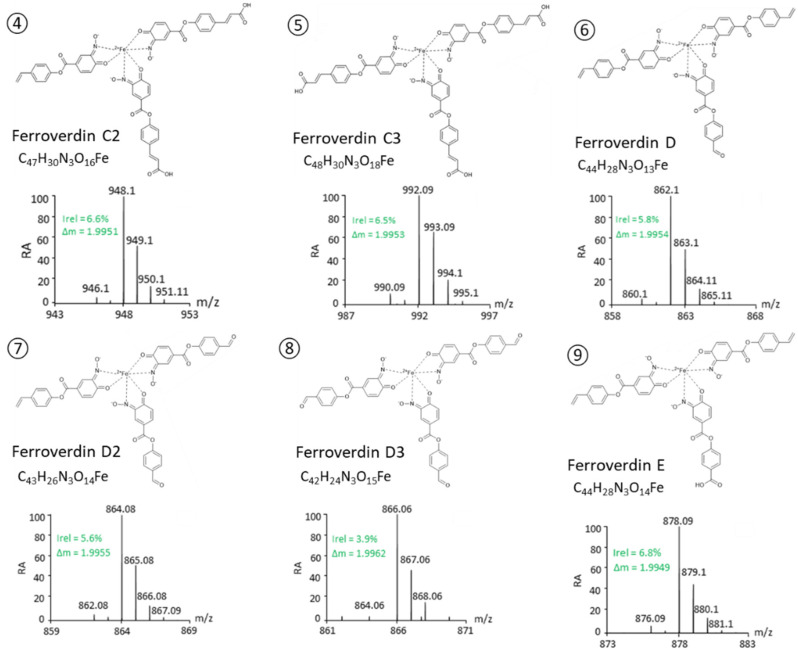

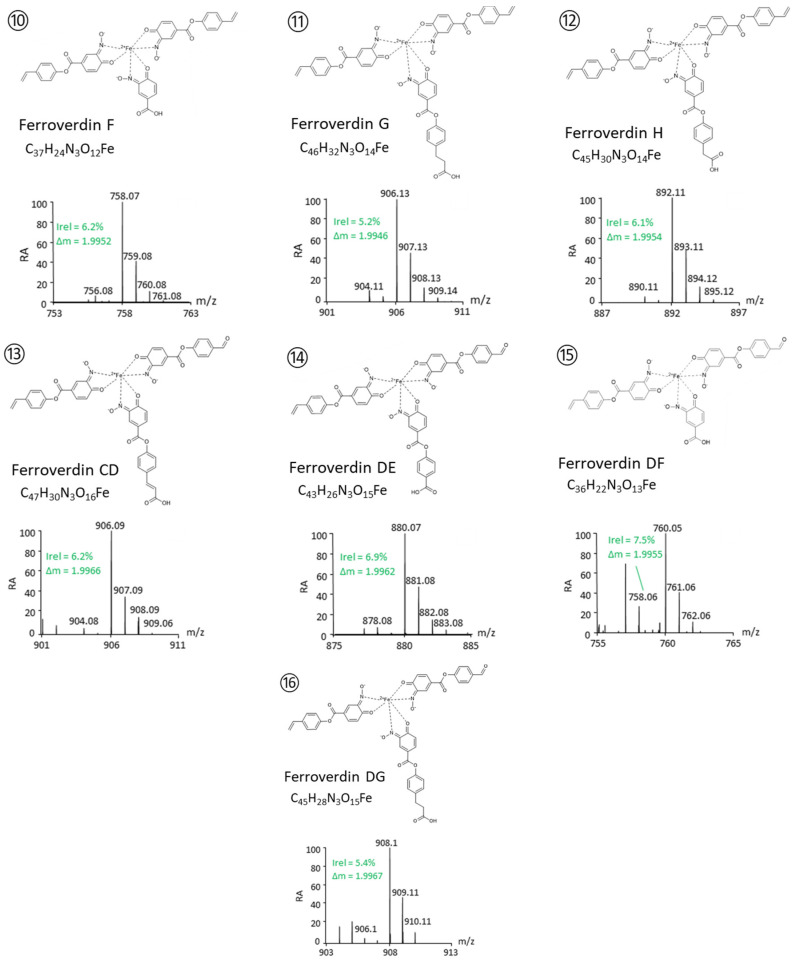
**Structure proposed for new ferroverdins.** Compound identification and structure elucidation was performed by UPLC–MS/MS. Each compound was identified based on its exact mass and isotope pattern and the analysis of the MS/MS spectra obtained by molecular ion fragmentation as detailed in supplementary Figure S1.

Based on these criteria, a total of 46 m/z ions were identified as possible ferroverdins (Table 1). For 13 of them, the fragmentation patterns allowed us to identify all 3 ferrous ion chelators. (See lines 4–16 in Table 1 and Figure 2).

**Table 1:** Ferroverdin-like compounds produced by the *S*. *lunaelactis* strains MM109^T^ and MM37.

#	Ferroverdin	Molecular Formula	*m/z* (Exp)	Δm(ppm)	Fe^2+^ Chelators	Reference
1	A	C_45_H_30_N_3_O_12_Fe^−^	860.1199	1.7	AAA	[8,9]
2	B	C_45_H_30_N_3_O_13_Fe^−^	876.1141	0.9	AAB	[8,9,10]
3	C	C_46_H_30_N_3_O_14_Fe^−^	904.1010	1.2	AAC	[8,9,10]
4	C2	C_47_H_30_N_3_O_16_Fe^−^	948.0990	1	ACC	This study
5	C3	C_48_H_30_N_3_O_18_Fe^−^	992.0868	1	CCC	This study
6	D	C_44_H_28_N_3_O_13_Fe^−^	862.0991	1.6	AAD	This study
7	D2	C_43_H_26_N_3_O_14_Fe^−^	864.0784	1.7	ADD	This study
8	D3	C_42_H_24_N_3_O_15_Fe^−^	866.0579	1.9	DDD	This study
9	E	C_44_H_28_N_3_O_14_Fe^−^	878.0933	0.7	AAE	This study
10	F	C_37_H_24_N_3_O_12_Fe^−^	758.0726	1.4	AAF	This study
11	G	C_46_H_32_ N_3_O_14_Fe^−^	906.1253	1.5	AAG	This study
12	H	C_44_H_30_N_3_O_14_Fe^−^	892.1101	2.1	AAH	This study
13	CD	C_45_H_28_N_3_O_15_Fe^−^	906.0888	1.3	ACD	This study
14	DE	C_43_H_26_N_3_O_15_Fe^−^	880.0732	1.5	ADE	This study
15	DF	C_36_H_22_ N_3_O_13_Fe^−^	760.0518	1.4	ADF	This study
16	DG	C_46_H_28_N_3_O_15_Fe^−^	908.1043	1.2	ADG	This study
From compounds 17 to 46, MS/MS fragmentation did not allow us to identify the third chelating molecule						
17	NA	C_47_H_32_N_3_O_15_Fe^−^	934.1196	0.79	AAX	This study
18	NA	C_39_H_30_N_3_O_10_Fe^−^	756.1295	1.2	AAX	This study
19	NA	C_38_H_31_N_3_O_16_Fe^−^	841.1068	1	AAX	This study
20	NA	C_45_H_32_N_3_O_14_Fe^−^	894.1246	0.8	AAX	This study
21	NA	C_47_H_32_N_3_O_13_Fe^−^	902.1298	0.8	AAX	This study
22	NA	C_54_H_56_N_6_O_19_Fe^−^	1148.2958	0.5	AAX	This study
23	NA	C_48_H_34_N_3_O_15_Fe^−^	948,1361	1.3	AAX	This study
24	NA	C_38_H_21_N_3_O_17_Fe^−^	857.1021	1.5	AAX	This study
25	NA	C_40_H_25_N_4_O_13_Fe^−^	825.0794	2.5	AAX	This study
26	NA	C_34_H_31_N_3_O_16_Fe^−^	793.0000	1.5	AAX	This study
27	NA	C_35_H_33_N_3_O_16_Fe^−^	807.0000	1.9	AAX	This study
28	NA	C_33_H_26_N_3_O_10_Fe^−^	680.0979	0.9	AAX	This study
29	NA	C_45_H_30_N_3_O_15_Fe^−^	908.0593	4.3	AAX	This study
30	NA	C_36_H_32_N_3_O_10_Fe^−^	722.1453	1.5	AAX	This study
31	NA	C_40_H_32_N_4_O_16_Fe^−^	880.11792	1.3	AAX	This study
32	NA	C_32_H_27_N_3_O_16_Fe^−^	765.0757	1.5	AAX	This study
33	NA	C_46_H_30_N_3_O_15_Fe^−^	920.1043	1.2	AAX	This study
34	NA	C_47_H_34_N_3_O_14_Fe^−^	920.1412	1.2	AAX	This study
For compounds 35 to 44, MS/MS fragmentation only identified *p*-vinylphenyl-3,4-NHBA as one of the three chelating molecules.						
35	NA	C_47_H_32_N_3_O_16_Fe^−^	950.1150	1.3	AXX	This study
36	NA	C_44_H_30_N_3_O_15_Fe^−^	896.1037	0.6	AXX	This study
37	NA	C_39_H_28_N_4_O_10_Fe^−^	768.1158	0.3	AXX	This study
38	NA	C_37_H_32_N_3_O_12_Fe^−^	766.1348	1	AXX	This study
39	NA	C_40_H_30_N_3_O_12_Fe^−^	800.1192	1	AXX	This study
40	NA	C_41_H_31_N_4_O_10_Fe^−^	795.1405	1.3	AXX	This study
41	NA	C_36_H_30_N_3_O_12_Fe^−^	752.1190	0.77	AXX	This study
42	NA	C_53_H_54_N_6_O_20_Fe^−^	1150.2754	0.2	AXX	This study
43	NA	C_43_H_26_N_3_O_15_Fe^−^	880.0732	1.5	AXX	This study
44	NA	C_40_H_30_N_5_O_16_Fe^−^	892.1035	0.1	AXX	This study
45	NA	C_35_H_30_N_3_O_10_Fe^−^	708.1296	1.4	?	This study
46	NA	C_35_H_28_N_3_O_10_Fe^−^	706.1140	1.4	?	This study

The letter(s) and number assigned to the newly structurally defined ferroverdins are based on the following principles: (1) a novel letter (starting from “D” as ferroverdins A, B, and C were previously designated) was given to ferroverdins that possess at least 1 unconventional molecule involved in iron chelation, in addition to *p*-vinylphenyl-3,4-NHBA; (2) the new letter was assigned according to the chronological order of its discovery (the first newly discovered molecule was assigned D, the second E, etc.); (3) the number associated with a letter (from 2 to 3) reflects the number of iron-chelating molecule(s) that are different from *p*-vinylphenyl-3,4-NHBA.

The proposed structures of these 13 new ferroverdins are displayed in Figure 2, and Table 2 lists all of the molecules involved in ferrous iron chelation in the 16 structurally elucidated ferroverdins. Tag signals of the MS/MS spectra of the molecular ion HCD fragmentation used for the identification of the molecules involved in ferrous iron chelation in novel ferroverdins are shown in Supplementary Figure S1. The remarkable features of the newly discovered ferroverdins are:

- A total of 5 novel ferroverdins (D(**6**), E(**9**), F(**10**), G(**11**), and H(**12**), Figure 2), as well as ferroverdin B(**2**) and ferroverdin C(**3**), also use 2 molecules of *p*-vinylphenyl-3,4-NHBA to bind to the ferrous iron, but the third molecule is an unconventional chelator: *p*-formylphenyl-3,4-NHBA for ferroverdin D(**6**), *p*-benzoic acid-3,4-NHBA for ferroverdin E(**9**), 3,4-NHBA for ferroverdin F(**10**), *p*-phenylpropionate-3,4-NHBA for ferroverdin G(**11**), and *p*-phenylacetate-3,4-NHBA(**12**) for ferroverdin H (see Figure 2 and Table 2).
- In 6 new ferroverdins, *p*-vinylphenyl-3,4-NHBA is only one of the 3 molecules used for ferrous iron binding: ferroverdins C2(**4**), D2(**7**), CD(**13**), DE(**14**), DF(**15**), and DG(**16**) (Figure 2). In the previously known ferroverdins, at least two molecules of *p*-vinylphenyl-3,4-NHBA were used for Fe^2+^ binding.
- Ferroverdins C3(**5**) and D3(**8**) (Figure 2) are remarkable as they are the first ferroverdins reported for which *p*-vinylphenyl-3,4-NHBA is never used for chelating Fe^2+^, but which are, instead, composed of 3 carboxy-*p*-vinylphenyl-3,4-NHBA, or 3 *p*-formylphenyl-3,4-NHBA, respectively.
- Remarkably, the 2 ferroverdins F(**10**) and DF(**15**) (Figure 2) recruit 3,4-NHBA for iron chelation; therefore, they are the only ferroverdins using a molecule not resulting from the activity of the FevW/BagE enzyme for the condensation of 3,4-NHBA with *p*-vinylphenol (see the proposed biosynthetic pathway for ferroverdin biosynthesis in [12]).

For 17 of the 43 novel ferroverdins (see Table 1, lines 17–34), some structural ambiguity remains for 1 of the 3 ferrous-iron-chelating agents, the two other molecules being *p*-vinylphenyl-3,4-NHBA. A total of 9 of the novel ferroverdin-like compounds present structural ambiguity for 2 of the 3 chelators, the known 1 being *p*-vinylphenyl-3,4-NHBA (see Table 1, compounds 35–44). Finally, 2 of the novel ferroverdin-like compounds present structural ambiguity for all the 3 ferrous-iron-chelating agents (see Table 1, compounds 45–46).

**Table 2:** Molecules involved in ferrous iron chelation in ferroverdins.

	Ferroverdins															
**Molecule involved in Fe^2+^ chelation**	1	2	3	4	5	6	7	8	9	10	11	12	13	14	15	16
	A	B	C	C2	C3	D	D2	D3	E	F	G	H	CD	DE	DF	DG
*p*-vinylphenyl-3,4-NHBA	3	2	2	1	-	2	1	-	2	2	2	2	1	1	1	1
Hydroxy-*p*-vinylphenyl-3,4-NHBA	-	1	-	-	-	-	-	-	-	-	-	-	-	-	-	-
Carboxy-*p*-vinylphenyl-3,4-NHBA	-	-	1	2	3	-	-	-	-	-	-	-	1	-	-	-
*p*-formylphenyl-3,4-NHBA	-	-	-	-	-	1	2	3	-	-	-	-	1	1	1	1
*p*-benzoic acid-3,4-NHBA	-	-	-	-	-	-	-	-	1	-	-	-	-	1	-	-
3,4-NHBA	-	-	-	-	-	-	-	-	-	1	-	-	-	-	1	-
*p*-phenylpropionate-3,4-NHBA	-	-	-	-	-	-	-	-	-	-	1	-	-	-	-	1
*p*-phenyacetate-3,4-NHBA	-	-	-	-	-	-	-	-	-	-	-	1	-	-	-	-

## 4. Discussion

In this work, we have demonstrated that the diversity of ferroverdin-like compounds is much broader than the three ferroverdins (A, B, and C) that had been previously reported. Indeed, ultra-performance liquid chromatography-high resolution mass spectrometry (UPLC–HRMS), in combination with tandem mass spectrometry (UPLC–MS/MS), allowed us to identify 43 novel ferroverdin-like compounds from the culture extracts of *S*. *lunaelactis* species. For 13 of these novel ferroverdins (newly designated Ferroverdins C2, C3, D, D2, D3, E, F, G, H, CD, DE, DF, and DG in this paper), analysis of their fragmentation pattern allowed us to identify the 3 molecules involved in Fe^2+^ binding. The chemical diversity of the ferroverdins only results from modifications of the *p*-vinylphenol parts of the ligands. This was expected since only the hydroxy and nitroso moieties of the ligands are involved in Fe^2+^ binding, which is best exemplified by ferroverdins F(10) and DF(15), in which 4-hydroxy-3-nitrosobenzoate (3,4-NHBA) is one of the 3 ligands (and is thus a ligand without the *p*-vinylphenol part added by the activity of FevW [10]).

Such a diversity of molecules involved in ferrous-iron chelation suggests that either (i) the additional biosynthetic genes in the *fev*/*bag* cluster would encode enzymes involved in the modification of Fe^2+^-binding molecules, (ii) a high substrate promiscuity of enzymes that will generate the *p*-vinylphenol derivatives (FevV, FevK, and FevL), and/or (iii) substrate promiscuity of FevW/BagE used to condensate 3,4-NHBA with the *p*-vinylphenol derivatives. At this stage, it is difficult to choose a more plausible explanation for the huge diversity of ferroverdins. Indeed, the enzymes of the proposed pathway have neither been enzymatically characterized regarding their substrate selectivity nor have their genes been inactivated to assess the impact on the accumulation of substrates and missing product(s). The fact that the *fev/bag* cluster is responsible for the production of both bagremycins and ferroverdins is already direct evidence that FevW/BagE is promiscuous. Indeed, from the proposed pathway, FevW/BagE will generate bagremycin A from the condensation of *p*-vinylphenol with 3,4-AHBA, whereas the same enzyme will produce the main monomer of ferroverdins (*p*-vinylphenyl-3,4-NHBA) from the condensation of *p*-vinylphenol and 3,4-NHBA [12]. However, for the new ferroverdins, it is *p*-vinylphenol that is replaced by other substrates for condensation with 3,4-NHBA by FevW/BagE. This was already the case for ferroverdin B and ferroverdin C, in which one of the three chelators is hydroxy-*p*-vinylphenol, and carboxy-*p*-vinylphenol, respectively. For the Fe^2+^ chelators newly identified in this study, FevW/BagE would use *p*-hydroxybenzaldehyde (in compounds 6, 7, 8, 13, 14, 15, and 16), *p*-hydroxybenzoic acid (in compounds 9 and 14), 3-(4-hydroxyphenyl)propanoic acid (in compounds 11 and 16), and *p*-hydroxyphenyl acetic acid (in compound 12) for condensation with 3,4-NHBA. Enzymatic in vitro studies with pure FevW/BagE and all of these candidate substrates (and other structurally similar substrates) should demonstrate the extent to which this enzyme displays substrate promiscuity for condensation with 3,4-NHBA. Alternatively, many “secondary” biosynthetic genes of the *fev*/*bag* cluster encode for oxygenase, dehydrogenase, and decarboxylase, and could thus be key enzymes involved in the modification of the ferroverdin molecules involved in ferrous-iron chelation.

Despite their diversity and high abundance when iron is provided in excess in the culture medium, the biological function of ferroverdins remains unknown. It was initially postulated that the function of ferroverdins would be to sequester the excess of Fe^2+^ in order to prevent damage to macromolecules from the reactive oxygen species generated by the Fenton reaction [10], but the production of ferroverdin levels does not correlate with the resistance of *S*. *lunaelactis* species to the toxic effect of iron overload (our unpublished data). Ferroverdins are structurally related to viridomycins, and viridomycin A, produced by a *Streptomyces* strain isolated from Moroccan phosphate mines, was recently shown to act as a rock phosphate solubilizer via its ability to chelate iron [34]. As *S*. *lunaelactis* strains have been isolated from cave moonmilk deposits [3,5,6,7], it is tempting to also attribute a possible role in rock solubilization to ferroverdins. However, ferroverdins are intracellular and not secreted like viridomycins, and therefore, their role in rock solubilization is unlikely. Also, moonmilk is present in limestone caves (calcium carbonate caves or calcium magnesium carbonate caves) and is not formed on phosphate rock. Therefore, if a small number of ferroverdins were to be released into the environment—due to cell death, for instance—they would not find phosphate rock in moonmilk speleothems.

Regarding their application, a patent for methods of ferroverdin production and their use as CETP inhibitors has been published [21]. With an IC_50_ value of 0.62 µM, ferroverdin B was reported as one of the most potent CETP-inhibitors of microbial origin [20]. The reason these hits have not passed the key preclinical or clinical stages of the drug discovery process (for example, production levels that are too low for large scale assays, cytotoxicity and/or failure of lead optimization, the strategic priority of more promising hits, etc.) is unknown. Our work revealed that the natural diversity of these molecules is much more important than initially thought, and some of these new compounds may be selected as candidate hits for the clinical development of CETP-inhibitors, or they may possess completely new biological activities.

## Data Availability

All data supporting the reported results and generated during the study are available in Figure S1, and in reference [4] regarding the methodology.

## Supplementary Materials

The following supporting information can be downloaded at: https://www.mdpi.com/article/10.3390/biom12060752/s1, Figure S1: Molecular tag signals for the identification of the ferrous-iron-chelating agents of novel ferroverdins.
