# Supplementary material for: Structure of New Ferroverdins Recruiting Unconventional Ferrous Iron Chelating Agents
Source: Biomolecules. 2022 May 26;12(6):752. doi: 10.3390/biom12060752 (PMC9221444; doi:10.3390/biom12060752)

Supplementary Figure S1. Molecular tag signals for the identification of the ferrous iron chelating agents of novel ferroverdins

④ **Ferroverdin C2**

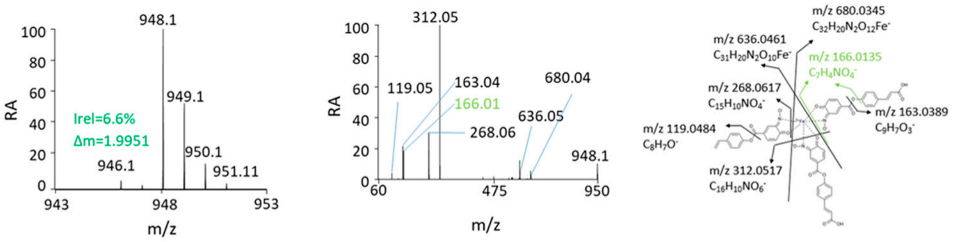

⑤ **Ferroverdin C3**

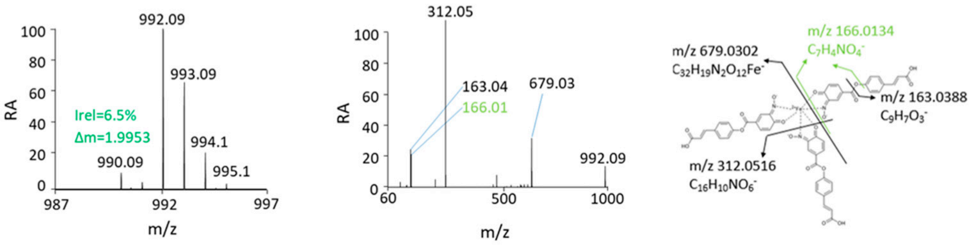

⑥ **Ferroverdin D**

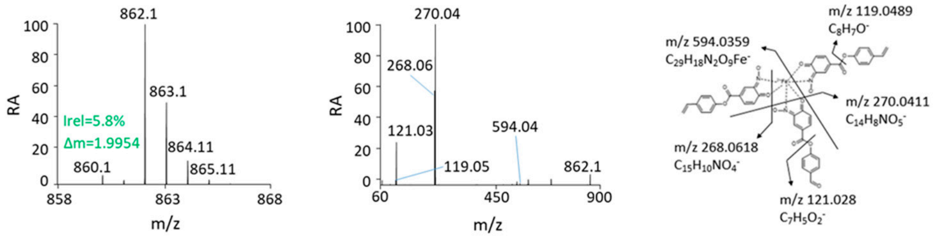

⑦ **Ferroverdin E**

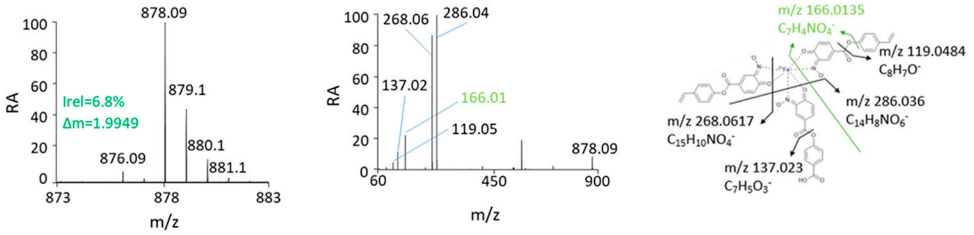

⑨ **Ferroverdin D3**

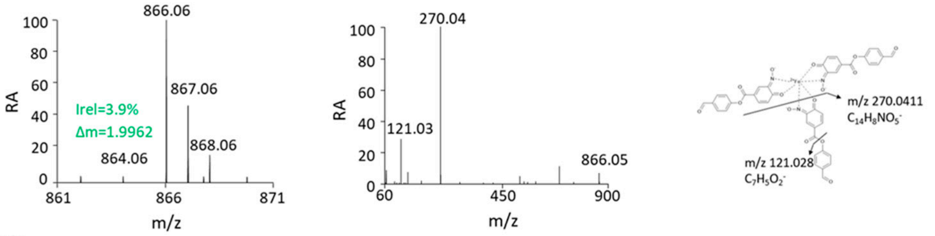

⑩ **Ferroverdin F**

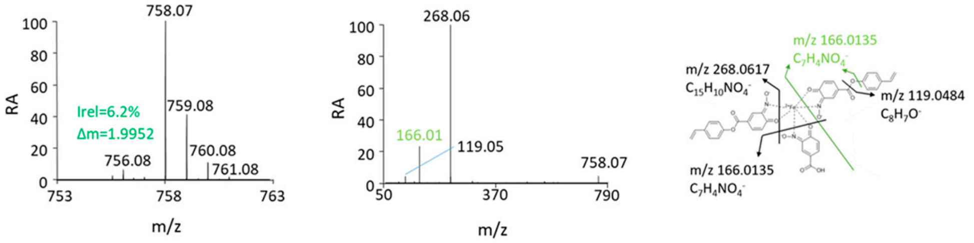

⑪ **Ferroverdin G**

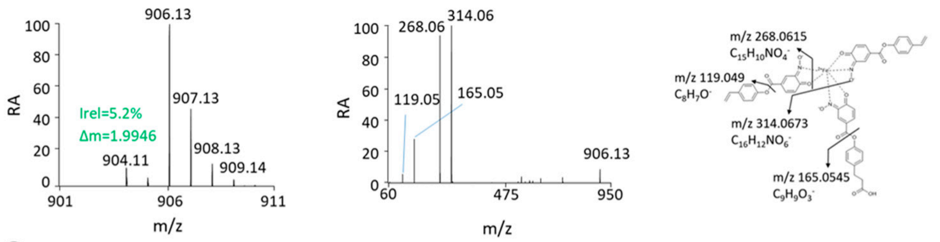

⑫ **Ferroverdin H**

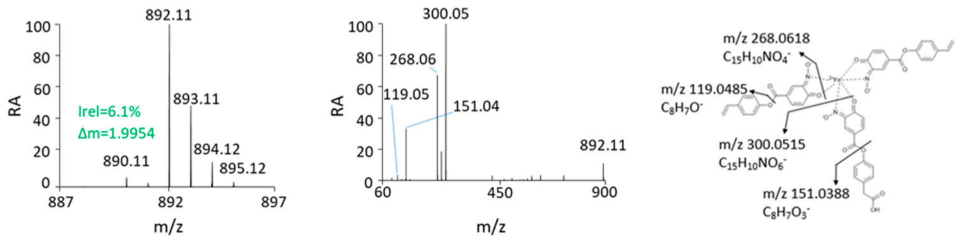

⑬ **Ferroverdin CD**

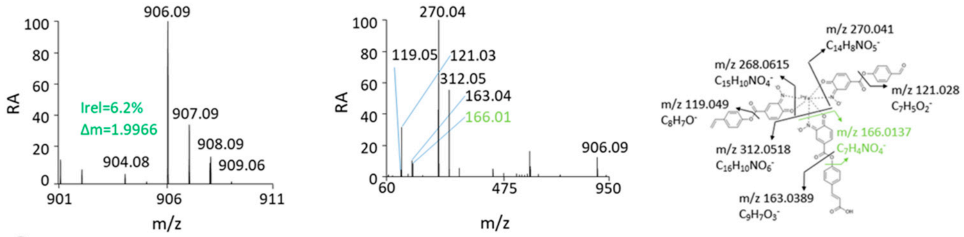

⑭ **Ferroverdin DE**

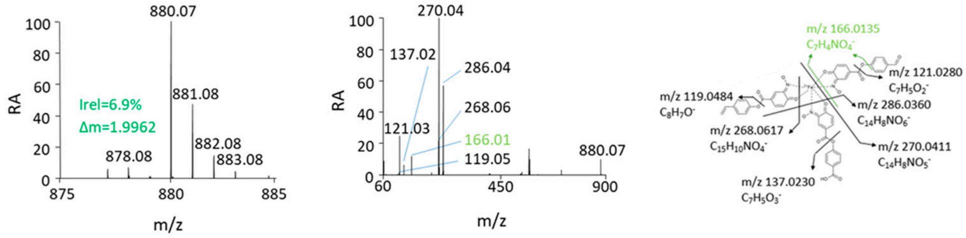

⑮ **Ferroverdin DF**

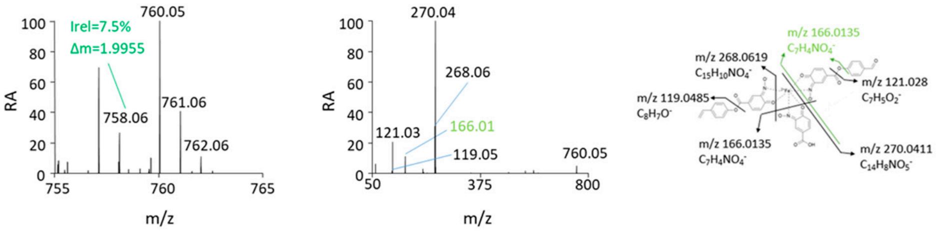

⑯ **Ferroverdin DG**

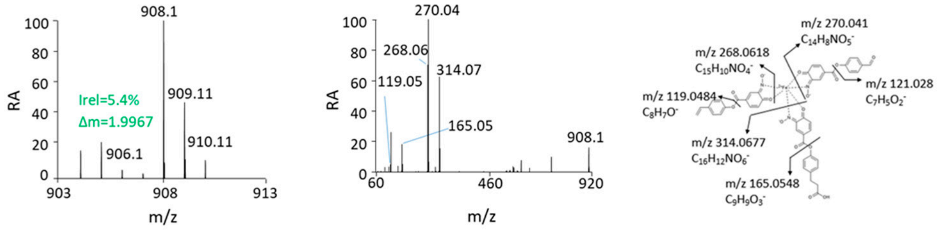

Supplement: Supplementary file 1 [file biomolecules-12-00752-s001.zip › biomolecules-1700543-supplementary.pdf]
